# Circular economy and environmental health in low- and middle-income countries

**DOI:** 10.1186/s12992-019-0501-y

**Published:** 2019-12-18

**Authors:** Caradee Y. Wright, Linda Godfrey, Giovanna Armiento, Lorren K. Haywood, Roula Inglesi-Lotz, Katrina Lyne, Patricia Nayna Schwerdtle

**Affiliations:** 1 Environment and Health Research Unit, South African Medical Research Council, 1 Soutpansberg Road, Pretoria, South Africa; 2 Department of Geography, Geoinformatics and Meteorology, University of Pretoria, Pretoria, South Africa; 3 Council for Scientific and Industrial Research, Pretoria, South Africa; 4 North-West University, Potchefstroom, South Africa; 5 ENEA Italian National Agency for New Technologies, Energy and Sustainable Economic Development, Rome, Italy; 6 Council for Scientific and Industrial Research, Pretoria, South Africa; 7 Department of Economics, University of Pretoria, Pretoria, South Africa; 8 Independant consultant, Adelaide, Australia; 9 Monash University, Nursing and Midwifery, Melbourne, Australia; 10 Heidelberg Institute of Global Health, University University, Heidelberg, Germany

**Keywords:** Circular economy, Environmental health, Low-and-middle-income countries, Sustainable production, Sustainable consumption, Sustainable development goals

## Abstract

**Background:**

The circular economy framework for human production and consumption is an alternative to the traditional, linear concept of ‘take, make, and dispose’. Circular economy (CE) principles comprise of ‘design out waste and pollution’, ‘retain products and materials in use’, and ‘regenerate natural systems’. This commentary considers the risks and opportunities of the CE for low- and middle-income countries (LMICs) in the context of the Sustainable Development Goals (SDGs), acknowledging that LMICs must identify their own opportunities, while recognising the potential positive and negative environmental health impacts.

**Main body:**

The implementation of the CE in LMICs is mostly undertaken informally, driven by poverty and unemployment. Activities being employed towards extracting value from waste in LMICs are imposing environmental health risks including exposure to hazardous and toxic working environments, emissions and materials, and infectious diseases. The CE has the potential to aid towards the achievement of the SDGs, in particular SDG 12 (Responsible Consumption and Production) and SDG 11 (Sustainable Cities and Communities). However, since SDG 3 (Good Health and Well-Being) is critical in the pursuit of all SDGs, the negative implications of the CE should be well understood and addressed. We call on policy makers, industry, the health sector, and health-determining sectors to address these issues by defining mechanisms to protect vulnerable populations from the negative health impacts that may arise in LMICs as these countries domesticate the CE.

**Conclusion:**

Striving towards a better understanding of risks should not undermine support for the CE, which requires the full agency of the public and policy communities to realise the potential to accelerate LMICs towards sustainable production and consumption, with positive synergies for several SDGs.

## Introduction

The World Health Organization (WHO) [1] explains that the circular economy (CE) is “… *a concept that focuses on closed loop material flows and the reduced consumption of virgin resources … (by)...changing models of consumption to maintain the highest value of materials and products and a change in utilisation patterns to extend product life.*” This is illustrated in the European Commission’s framework for the CE comprising production, consumption, waste management and ‘from waste to resources’ whereby ‘secondary raw materials’ can be used just as new materials [2, 3]. The CE concept for human production and consumption – ‘renew, remake, and share’ – has been proposed widely as an alternative to the traditional linear concept – ‘take, make, and dispose’ – to minimise resource input, waste, emissions, and energy leakage through reduce, remanufacture, repair, renew, reuse, and recycle principles [4]. These principles are applied holistically from resource extraction, to parts and product manufacturing, consumer use and then into a cascade of share, maintain, reuse, redistribute, refurbish, and recycle activities. For example, substitution of finite, non-renewable resources with renewable resources enhances natural capital stocks and balances renewable resource flows [1]. Here, we consider the risks and opportunities of the CE for low- and middle-income countries (LMICs), acknowledging that LMICs must identify their own opportunities, and recognising the potential positive and negative environmental health impacts of CE. We also consider positive synergies related to CE implementation and achievement of several SDGs when an intersectoral approach is adopted.

## Circular economy in the context of developing countries

Within LMICs countries, the CE has been identified as an opportunity to progress towards sustainable development, resource efficiency, and a low-carbon economy. Developed countries, particularly those in the European Union (EU), are driven largely by issues of resource constraints and have consequently widely adopted the CE concept into policy [1]. The CE has found traction within developed countries; however, this is not the case in LMICs that are still largely coming to terms with the CE concept and its national relevance. Significant gaps persist in the understanding of whether adoption of CE practices in the ‘Global South’ will contribute positively towards economic growth, jobs, and sustainable development. However, it is important to acknowledge that lower-income countries are in many ways already more ‘circular’ than their developed-economy counterparts. A CE is often the default economy in a low-income setting because of lower levels of consumption and lesser availability of material goods [5]. The question is how to turn CE into a development opportunity [4] and how to protect and promote health in the transition of its realisation. To date, the implementation of the CE in LMICs has mostly been undertaken informally, driven mainly by poverty and unemployment, and includes activities such as recycling, repair, and reuse. As such, there are many missed opportunities for cleaner production; remanufacturing, product sharing; increased responsibility and awareness among producers and consumers; the use of renewable technologies and materials; and the adoption of appropriate policies and tools.

While the key drivers for adopting CE principles in developed countries include resource security and environmental sustainability, for LMICs, the drivers may include ‘extracting value’ from waste as secondary resources that can be used to create livelihoods, generate jobs, and reduce poverty. Ironically, it is in these and related activities that environmental health risks exist. It is critical, while unpacking the opportunities that the CE provides for LMICs, to consider the potential positive and negative environmental health impacts. This is particularly relevant for LMICs given the large, active informal sector and the labour-intensive approach adopted by government and business, as well as the relative lack of regulation to protect workers’ health. In addition, the CE may help deal with the pressures of industrialisation and urbanisation as LMICs are facing a growing waste crisis, which has major consequences for environmental and health outcomes [6].

## Environmental health perspective

Environmental health aims to prevent adverse impacts on human health from all environmentally derived factors (such as waste, water, and air pollution), and create health-supportive environments. “*Environmental health addresses all the physical, chemical, and biological factors external to a person, and all the related factors impacting behaviours …*. *(and) … it encompasses the assessment and control of those environmental factors that can potentially affect health”* [5]. The successful use of the CE principles within economic planning and policymaking is dependent on an achievement of net gains, ensuring that positive effects outweigh the negative impacts for the environment, and the health and living standards of society.

**Fig. 1:**
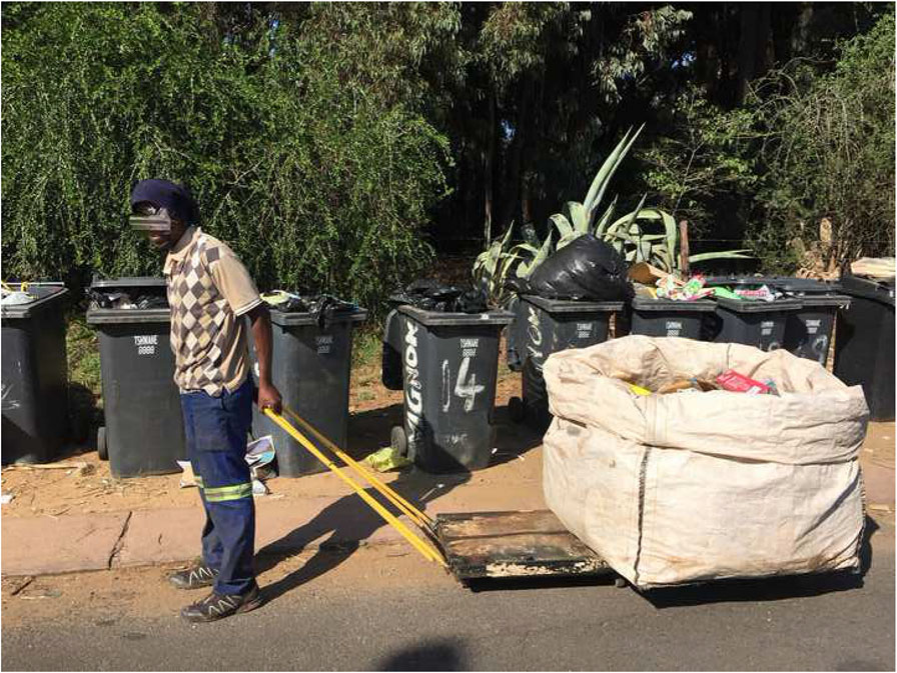
Informal recycler working at kerbside in Pretoria, South Africa (Photographer: CY Wright)

In August 2018, The WHO European Centre for Environment and Health published the report “*Circular Economy and Health: Opportunities and Risks*” which evaluates the human health impacts of CE activities [1]. The report recognises that the CE creates opportunities for improved environmental health, but also highlights the potential for negative impacts. For example, transitioning to a CE will likely contribute directly towards savings in the healthcare sector from reduced environmental pollution and associated illnesses, but also unintended adverse health effects from exposures to hazardous materials. In LMICs where CE activities are largely informal, particularly in the early stages of the value chain, a wide range of environmental health impacts may occur. These include exposure to hazardous working conditions, emissions, and materials i.e. exposure to toxic fumes when burning tyres or electronic waste to extract metal. There are also several risks for informal waste pickers working at kerbsides (Fig. 1) and dumpsites, including exposure to methane, mould and airborne (fungal) spores and subsequent respiratory and skin infections [6]. Additionally, there is risk of needle stick injuries, exposure to human excrement and body fluids, consuming contaminated food, and increased risk of infectious diseases such as malaria, zika, cholera, hepatitis, and others [7]. On 20 February 2018, 17 people living alongside a dumpsite in Mozambique lost their lives while foraging for food and sellable items when the dumpsite collapsed [8]. Dumpsites account for a large share of global greenhouse gas emissions [9], and many people per year are exposed to dangerous concentrations of lead at battery recycling sites [10] or die prematurely due to the open burning of waste [6]. For example, in 2007 there were reports of high child mortality in Dakar, Senegal, from acute lead poisoning which was linked to the recycling of used lead-acid batteries [11]. These risks frequently burden mostly vulnerable groups – women, children [11], low-income groups and the informal sector – and highlight the urgency of finding new ways to meet development goals while reducing resource consumption [4].

## Opportunities

There is limited understanding of what the CE means for LMICs, consequently there is a paucity of research that analyses the potential impacts of CE on human health. On a positive note, at a side event of the 23rd Conference of the Parties to the United Nations Framework Convention on Climate Change, the *African Alliance on the Circular Economy* was launched [12]. The Alliance is a union between the governments of Rwanda, Nigeria, and South Africa in conjunction with World Economic Forum (WEF) and the Global Environment Facility (GEF), and is responsible for fast tracking the adoption of the CE and other partnerships required to meet the SDGs. They have identified that the potential to generate wealth from waste, especially among poor, marginalised communities, is deemed a significant opportunity by many LMICs’ governments. The need for an intersectoral approach is recognised by the Alliance, which aims to build synergies between the economy, environment, and society. Another opportunity is offered by the African Circular Economy Network (ACEN) which is more of a non-state advocacy initiative. The ACEN aims to build a restorative African economy that inclusively generates well-being and prosperity through new forms of economic production and consumption, whilst preserving and regenerating environmental resources.

Despite these latent socio-economic and environmental gains, caution and public debate are needed to fully consider the environmental health threats that vulnerable groups, especially women and children, may face in all elements of CE implementation. This is particularly pertinent where environment and health linkages exist, given the existing precarious nature of this relationship in LMICs. For example, reprocessing of dumped e-waste has had devastating impacts on communities and the environment; battery acid leaching and toxic gas inhalation caused by burning of wire insulation for metal recovery illustrates this impact [13, 14]. There have been measures put in place to reduce risks for people living off waste; for example, in Pune, India, the municipal administration institutionalised door-to-door waste collection, instead of on landfills, and provided health and safety equipment for waste pickers in the SWaCH (Solid Waste Collection and Handling) Cooperative [15]. This example highlights the importance of an intersectoral approach to implementing the CE: to optimise waste management opportunities while protecting human health.

Critics align in identifying a key flaw of the SDGs being the assumption that the economic system that created current levels of unprecedented inequality can be used to engineer the reverse [16]. It seems a transformation of the system, such as through the adoption of the CE, is more logically aligned with the achievement of the SDGs: more directly SDG12 – Responsible Consumption and Production – and SDG11 – Sustainable Cities and Communities. Strong links can be found between CE applications and other SDGs such as SDG 6 (Clean Water and Sanitation), SDG 7 (Affordable and Clean Energy), and SDG 15 (Life on Land) [17]. CE can indirectly create synergies and accelerate the achievement of targets, such as promoting economic growth and jobs (SDG 8), elimination of poverty (SDG 1), and ending hunger (SDG 2). While SDG 8 (Decent work and economic growth) and SDG 9 (Industry, Innovation and Infrastructure) may initially seem threatened by the CE concept, with more research and sufficient consideration, progress towards these goals have the potential to be boosted by the CE. Since SDG 3 (Good Health and Well-being) is recognised as critical for the achievement of all the other SDGs, it is imperative that the influences of the CE on environmental health be considered on the path to sustainable development [18]. Whilst critics highlight the incompatibility of continued socio-economic development and environmental sustainability, models identify some factors capable of contributing to development (SDG 3) on one hand whilst contributing to economic sustainability (SDG 7) on the other [19], the CE being a good example. Policies should be cautious of potential trade-offs of promoting, for instance, recycling of household waste in lieu of human health (SDG 3.9). The CE could transform human perceptions and behaviours regarding production and consumption with potential benefits for environmental and human health. The use of clean, renewable energy sources for household lighting in sub-Saharan Africa is one example of reducing resource consumption in low-income settings [20]. There are, however, barriers to and potential risks involved in the transition, particularly for LMICs. We call on policy makers, industry and the health and health-determining sectors to protect populations, especially vulnerable populations, from potential negative health impacts that may arise as countries domesticate the CE. Cross-sectoral and intersectoral partnerships, as called for by SDG 17 (Partnerships for the Goals) will facilitate the implementation of the CE while promoting health and sustainable development.

## Conclusions

Striving towards a better understanding of potential risks should not undermine support for the CE, which requires the full agency of the public and policy-making communities to realise the potential for LMICs to accelerate towards sustainable production and consumption with positive synergies for many other SDGs. To our knowledge, there is no comprehensive and reliable research available regarding the costs and benefits of a CE on environmental health in LMICs. Importantly, CE strategies could be a means for LMICs to ‘leapfrog’ to a more sustainable development pathway with social, economic, and health co-benefits. The transformation of plastic waste into retail opportunities, such as turning tyres into shoes [18], is already occurring in several regions in Africa. While the CE does create opportunities for LMICs, it is important to find a tailored means to do so, in other words, to domesticate the CE principles and recognise that a clear understanding of the potential health impacts (both positive and negative) is needed. As countries develop this understanding, it is imperative that the environmental health implications be considered, and, where necessary, policies and actions be put in place to mitigate the potential negative health impacts.

## Data Availability

Data sharing is not applicable to this article as no datasets were generated or analysed during the current study.

## Abbreviations

CE: Circular economy
EU: European Union
LMICs: Low-and middle-income countries
SDGs: Sustainable Development Goals
WHO: World Health Organization
